# Combined Transcriptomic and Proteomic Profiling Uncovers Developmental Dynamics of Autophagy in the Cortex

**DOI:** 10.3390/biomedicines14040812

**Published:** 2026-04-02

**Authors:** Francesca Nuzzolillo, Clarissa Braccia, Annapaola Andolfo, Stefano de Pretis, Michela Palmieri

**Affiliations:** 1 Faculty of Medicine and Surgery, Vita-Salute San Raffaele University, 20132 Milan, Italy; nuzzolillo.francesca@hsr.it; 2 ProMeFa, Proteomics and Metabolomics Facility, IRCCS San Raffaele Scientific Institute, 20132 Milan, Italy; braccia.clarissa@hsr.it (C.B.); andolfo.annapaola@hsr.it (A.A.); 3 Center for Omics Sciences, IRCCS San Raffaele Scientific Institute, 20132 Milan, Italy; depretis.stefano@hsr.it; 4 Rett Syndrome and Neurodevelopmental Disorders, Division of Neuroscience, IRCCS San Raffaele Scientific Institute, 20132 Milan, Italy

**Keywords:** autophagy, lysosome, cortical development, synapsis, transcriptomic, proteomic

## Abstract

**Background/Objectives:** Autophagy is an evolutionarily conserved degradation and recycling pathway through which cells deliver cytoplasmic components, including toxic or damaged proteins and organelles, to lysosomes for clearance. In neurons, which are largely post-mitotic, degradative pathways are essential to prevent the accumulation of cellular waste and to maintain nutrient and energy homeostasis. Increasing evidence suggests that autophagy plays a critical role during early brain development, when neuronal circuits are established, synaptic connections are refined, and activity-dependent mechanisms shape network architecture. However, the developmental regulation of autophagy-related genes and the composition of the autophagic machinery at synapses remain poorly understood. This study aimed to characterize the maturation-dependent dynamics of autophagy–lysosomal genes and to investigate the synaptic autophagy-associated proteome during cortical development. **Methods:** Genome-wide transcriptomic analyses were performed in the cortical brain region across developmental stages to assess changes in the expression of autophagy–lysosomal genes. In parallel, synaptosomes were isolated and subjected to proteomic analysis to identify autophagy-related proteins associated with synaptic compartments. **Results:** Transcriptomic profiling revealed stage-dependent regulation of autophagy–lysosomal genes during cortical maturation. Proteomic analysis of synaptosomes identified multiple autophagy-associated proteins enriched at synaptic sites, suggesting that components of the autophagic machinery are present at synapses and may participate in synaptic remodeling and function during key phases of neuronal network formation. **Conclusions:** These findings provide new insights into the developmental regulation of autophagy in the brain and highlight the potential contribution of synaptic autophagy to neuronal circuit maturation. Understanding these mechanisms may help identify novel therapeutic targets for neurological disorders associated with impaired synaptic and cellular homeostasis.

## 1. Introduction

Macroautophagy (hereafter referred to as autophagy) is an evolutionarily conserved process devoted to the elimination of detrimental cellular components like dysfunctional organelles, toxic macromolecules, and unfolded proteins [1]. It is particularly important in neurons, which are post-mitotic cells, metabolically very active, and highly vulnerable to any loss of cellular homeostasis [2]. Consistent with this, defective autophagy function has been linked to aging and neurodegenerative diseases [3,4,5]. Interestingly, increasing evidence also indicates that autophagy is involved in multiple stages of neurodevelopment, including neuronal differentiation, neurite overgrowth, neurite guidance, and the formation and refinement of synaptic connections. For instance, loss of the autophagy-related gene 5 (*Atg5*) in dividing neural progenitor cells (NPCs) within the adult hippocampus led to reduced autophagic flux and delayed neuronal maturation [6]. Similarly, deletion of *ATG7* in hypothalamic proopiomelanocortin (POMC) neurons caused loss of axonal projections, highlighting the essential role of autophagy in supporting axonal growth in vivo [7]. Moreover, disruption of core autophagy genes in rodent neurons has been shown to alter multiple aspects of synaptic function, including synaptic maturation, assembly, neurotransmitter release, and neuronal plasticity [8,9,10]. Furthermore, autophagy may regulate synapses by directly degrading key synaptic protein substrates. Post-synaptic scaffolds PSD-95, PICK1, and SHANK3, which are crucial for dendritic spine remodeling, were identified as autophagic cargos [8]. Finally, mutations in genes encoding for regulators and components of the autophagic-lysosomal system have been linked to neurodevelopmental disorders and childhood-onset neurological diseases [11,12,13,14]. All these findings suggest that autophagy regulates neuronal maturation and synaptic machinery at different levels; however, how it modulates all these processes is still unclear.

Recent omics-based studies, particularly RNA sequencing coupled with pathway enrichment analyses, have been extensively used to investigate autophagy, largely in the context of pathological conditions, highlighting the strong association between autophagy dysregulation and disease onset or progression. Alongside these advances, the identification of the transcription factors TFEB and TFE3, members of the basic helix–loop–helix leucine zipper family, has revealed the existence of a transcriptional program that coordinates the expression of autophagy and lysosomal genes [15,16,17]. Despite these important discoveries, most transcriptomic and bioinformatic analyses have focused on disease-related or stress-induced autophagy. Consequently, the regulation of autophagy under basal physiological conditions—particularly during brain development—remains largely unexplored. To investigate this, we performed a comprehensive transcriptomic analysis of the cortical brain region to define the core set of autophagy–lysosomal genes expressed throughout distinct stages of brain maturation, including neurogenesis, axon and dendritic growth, and synaptogenesis. To elucidate how autophagy contributes to synaptic function, we also characterized the proteomic landscape of synaptosomes, focusing on the autophagy-associated proteins that operate at developing synapses. These findings advance our understanding of how neuronal autophagy is developmentally regulated and whether it contributes to the establishment and maintenance of synaptic connectivity. Moreover, this work provides novel insight into mechanisms that may explain synaptic vulnerability in neurodevelopmental disorders and highlights potential therapeutic targets for these conditions.

## 2. Materials and Methods

### 2.1. Animals

Wild-type (WT) male and female mice (Mus musculus) were obtained from Charles River Laboratories (Calco, Italy) and housed in an SPF facility (IRCCS San Raffaele Scientific Institute, Milan, Italy), with a 12 h light/dark cycle and food and water ad libitum. All experiments involving animals were performed with authorization from the Italian Ministry of Health, according to international guidelines for animal welfare (European Directive 2010/63/EU).

### 2.2. Total RNA Extraction

Total RNA was extracted from the cerebral cortices of wild-type (WT) mice at embryonic day 16 (E16), postnatal day 5 (P5), and postnatal day 10 (P10) using the Qiagen RNeasy Mini Kit (74104), (Qiagen, Hilden, Germany), following the manufacturer’s instructions. Immediately after dissection, tissues were snap-frozen on dry ice and stored at −80 °C until RNA extraction. On-column DNase I digestion was performed to eliminate residual genomic DNA. RNA concentration and purity were measured using a NanoDrop 1000 spectrophotometer (Thermo Fisher Scientific, Monza, Italy), and RNA integrity was assessed using an Agilent 2100 Bioanalyzer, Agilent Technologies, Santa Clara, CA, USA. Only samples with an RNA Integrity Number (RIN) ≥ 8 were used for downstream analyses.

### 2.3. Real-Time qPCR

Total RNA from six males and six females at each time point (E16, P5, and P10) was extracted and reverse transcribed using the QuantiTect Reverse Transcription Kit (Qiagen, 330404), starting from 1 µg of total RNA. Gene expression was analyzed by real-time PCR (qPCR) using a SYBR Green-based Master Mix (Thermo Fisher Scientific, 4364346). Reactions were performed in technical triplicates under standard amplification conditions, followed by a melting curve analysis to assess product specificity. Relative gene expression was calculated using the ΔΔCt method, with *cyclophilin* as the housekeeping gene. Data analysis was carried out using GraphPad Prism9.0 (Graphpad Software Inc., La Jolla, CA, USA) and differences were considered statistically significant at *p* < 0.05. The primers listed in Table 1 were used for real-time qPCR validation of autophagy–lysosomal genes selected from curated gene lists elaborated by Bordi and colleagues. These genes, representing autophagy regulators, lysosomal components, and core autophagy machinery, were analyzed in both male and female mice.

### 2.4. RNAseq and Data Processing

Total RNA was extracted from five to eight male and female WT cortices pooled together. Only high-quality RNA with an RNA Integrity Number (RIN) of 9 or higher was used. Library preparation and sequencing were performed with TruSeq RNA Library Preparation Kit v2 (Illumina Novaseq, Illumina, San Diego, CA, USA) by Genewiz. FASTQ sequencing reads were adapter-trimmed and quality-filtered with Trimmomatic [18] prior to mapping to the mm10 mouse reference genome: Genecodegenes.com v.M22 accessed 4 December 2024 (https://www.gencodegenes.org/mouse/) with STAR [19]. Gene counts were obtained using featureCounts [20]. Normalization and differential gene expression analysis (DEG) were performed with DESeq2 [21]. Only genes with sufficient expression levels were included in the DGE testing. Specifically, 9211 genes exhibiting an average expression of at least 5 FPKM and a minimum of 10 counts in at least one of the three profiled conditions (E16, P5, or P10) were considered for analysis. Heatmap visualization and clustering were performed using the R package pheatmap v.4.1.1. Gene ontology enrichment analysis and GSEA were performed using the R package clusterProfiler v.4.0.5 [22]. For Gene Ontology (GO) enrichment, the Biological Process (BP) ontology was interrogated using the genes belonging to each transcriptional cluster, which were analyzed separately. Enrichment *p*-values were adjusted for multiple testing using the Benjamini–Hochberg method to control the false discovery rate (FDR). The five most significantly enriched biological processes for each cluster (adjusted *p* < 0.05) were visualized using a dot plot.

### 2.5. Synaptosome Enrichment Procedure

Synaptosomes were isolated from the cerebral cortices of wild-type mice at postnatal days P5, P10, and P20, with three to four biological replicates per time point. For each replicate, half of the cortex from a male mouse and half from a female mouse were pooled. The extraction buffer was prepared using 0.32 M sucrose and 4 mM HEPES, supplemented with protease (04693132001, Merck, Darmstadt, Germany) and phosphatase inhibitors (4906845001, Merck, Germany) to preserve protein integrity. Cortical tissue was mechanically homogenized on ice using a Potter-Elvehjem homogenizer (Fisher Scientific, Monza, Italy). The homogenate was subjected to differential centrifugation as follows: 800× *g* for 3 min at 4 °C to remove nuclei and debris, followed by 800× *g* for 15 min at 4 °C, and finally 10,200× *g* for 15 min at 4 °C. The resulting pellet, containing the synaptosomal fraction, was carefully collected and resuspended in lysis buffer containing 1% SDS for downstream Western blot and proteomic analysis. Protein concentration of the synaptosomal lysates was quantified using the bicinchoninic acid (BCA) assay (Pierce™ BCA Protein Assay Kit, 23227, Thermo Fisher Scientific, Italy), following the manufacturer’s instructions. Equal amounts of protein (30 µg) were then processed for label-free quantitative proteomics.

### 2.6. Western Blotting

Protein lysates were separated by SDS-PAGE. Briefly, 10 µg of protein per sample was loaded onto 10% (TGX™ FastCast™ Acrylamide Kit, 1610175, Biorad, Segrate, Italy) or 12% gels (TGX™ FastCast™ Acrylamide Kit, 1610173, Biorad, Italy) and run in running buffer (25 mM Tris, 192 mM glycine, 0.1% SDS) at 100 V. Proteins were transferred onto nitrocellulose membranes using a semidry transfer system (Trans-blot Turbo Transfer system, 704150, Bio-Rad, Segrate, Italy) according to the manufacturer’s instructions. Blocking and incubation with primary and HRP-conjugated secondary antibodies (Jackson ImmunoResearch, West Grove, PA, USA) were performed in 5% milk in TBST (150 mM NaCl, 20 mM Tris-HCl, pH 7.6, 0.1% Tween-20). Primary antibodies used in this study are: anti-Rab5a (SC-46692, Santa Cruz Biotechnology, Dallas, TX, USA), anti-Rab11b (PA531348, Thermo Fisher Scientific, Italy), anti-Atp6v0l (NBP1-89342, Bio-techne, Minneapolis, MN, USA), anti-Atp6v1a (MA527730, Thermo Fisher Scientific, Italy), anti-Synapsin1 (106002, Synaptic Systems, Göttingen, Germany), anti-PSD95 (124011, Synaptic Systems, Germany), anti-VGLUT1 (135302, Synaptic Systems, Germany), anti-VGAT1 (131003, Synaptic Systems, Germany), and anti-VAMP2 (104211, Synaptic Systems, Germany). Chemiluminescent detection was carried out using a Chemidoc MP imaging system (12003154, Bio-Rad, Italy) with Western Sun (XLS063, Cyanagen, Italy) or Western Antares (XLS141, Cyanagen, Bologna, Italy) substrates.

### 2.7. Sample Preparation for Proteomic Analysis

Proteins were digested following the FASP (Filter Aided Sample Preparation) protocol [23]: disulfide bonds of cysteines were reduced with an incubation at 95 °C for 1 min with 50 µL of 0.1 M DTE (1,4-dithioerythritol) in Tris/HCl pH 7.4. After two centrifugation steps on Amicon Ultra-3K at 14,000× *g* for 10 min with a solution of 8 M urea, cysteine residues were alkylated with 100 µL 0.05 M IAA (iodoacetamide) in 8 M urea. Samples were centrifuged twice on Amicon Ultra-3K at 14,000× *g* for 10 min with a solution of 8 M urea and then incubated overnight at 37 °C in the presence of sequencing grade trypsin (Sigma-Aldrich, St. Louis, MO, USA) 1:50 (*w*/*w*), upon dilution of urea up to 2 M with 50 mM ammonium bicarbonate buffer. The obtained peptides were subsequently desalted using homemade Stage Tips C18.

### 2.8. LC-MS/MS Analysis

Desalted peptides were injected into a capillary chromatographic system (Vanquish™ Neo UHPLC System, Thermo Fisher Scientific) for peptide separations on a 75 µm i.d. × 15 cm reverse-phase silica capillary column, packed with a 1.9 μm ReproSil-Pur 120 C18-AQ (Dr. Maisch GmbH, Ammerbuch-Entringen, Germany). A 100 min gradient of eluents A (pure water with 0.1% *v*/*v* formic acid) and B (acetonitrile with 0.1% *v*/*v* formic acid) was used to achieve separation (from 3% to 29% of B in 88 min, 300 nL/min flow rate). MS analyses were performed using a Q-Exactive mass spectrometer (Thermo Scientific, Bremen, Germany) equipped with a nano-electrospray ion source (Proxeon Biosystems). Each sample was analyzed in technical triplicate for label-free quantitative proteomics. Full scan spectra were acquired with the lock-mass option, resolution set to 70,000, and mass range from *m*/*z* 300 to 2000. The ten most intense ions (charge exclusion: unassigned, 1, 6–8, >8) were selected to be fragmented (ddMS2). MS/MS spectra were acquired with resolution set to 17,500, NCE set to 25, and an isolation window of 2 *m*/*z*.

### 2.9. Data Processing and Statistical Analysis

Raw data were then analyzed by MaxQuant software (v. 1.6.1.0) [24] for label-free protein quantification based on the precursor intensity. Searches were performed with the following settings: trypsin as proteolytic enzyme; two missed cleavages allowed; carbamidomethylation on cysteine as fixed modification; protein N-terminus acetylation and methionine oxidation as variable modifications; mass tolerance was set to 5 ppm and to 20 ppm for precursor and fragment ions, respectively; mouse_proteome 20220525 (63,641 sequences; 28,558,480 residues) was used as database. An FDR (false discovery rate) of 1% was selected for both peptide and protein identification, with a minimum of two peptides per protein with at least one unique peptide. The complete dataset of identified and quantified proteins was processed using an in-house developed R script as follows: log transformation; filtering for proteins with at least a 50% quantification rate in one or more conditions; imputation of missing values. ANOVA post hoc with Tukey correction was performed to detect the dysregulated proteins. GO (Gene Ontology) analyses were performed using David software (v.6.8), freely available [25], setting a list of proteins known to be expressed in the brain. The alluvial plot was generated by categorizing the 37 significantly deregulated autophagy–lysosomal proteins into the functional groups defined by Bordi et al. and visualizing their distribution across categories using an in-house R script (available upon request). Hereafter, the term “deregulated” is used to refer to proteins whose abundance significantly changes across developmental stages, as determined by our proteomic analysis. These changes reflect the dynamic regulation of protein levels during cortical maturation and do not necessarily imply functional impairment or “dysfunction.”

## 3. Results

### 3.1. Identification of Distinct Differential Expression Patterns and Functional Gene Clusters in the Developing Cortex

**Figure 1:**
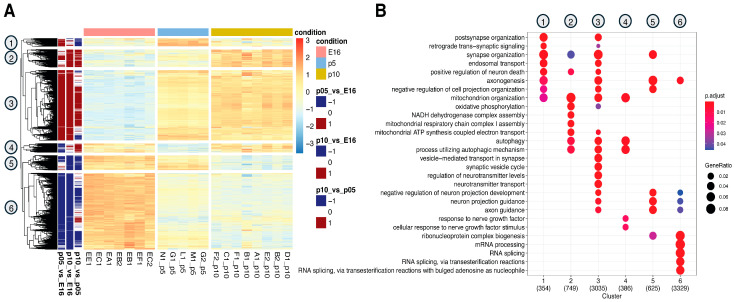
Coordinated pathway networks drive cortical development. (**A**) Heatmap showing the relative transcript levels of all differentially expressed genes (DEGs, rows) across developmental stages (columns). Columns are grouped into three major developmental time points: E16, P5, and P10. Red indicates high gene expression, and blue indicates low expression. (**B**) Dot plot depicting the biological processes enriched within the six clusters (numbered 1 to 6) identified by hierarchical clustering of the expression data (*p* adj < 0.05).

To define the transcriptomic profile of genes deregulated during early phases of cortical development, we extracted total RNA from female and male littermates at E16 (embryonic day 16), P5 (postnatal day 5), and P10 (postnatal day 10), corresponding to the peak of neurogenesis, neuronal differentiation and early synaptogenesis, and synaptic maturation with circuit refinement, respectively. RNA samples were then subjected to sequencing using the Illumina NextSeq6000 system and analyzed to identify differentially expressed genes. Principal Component Analysis (PCA) revealed a clear separation among the three different time points (Supplementary Figure S1A), indicating significant changes in the transcriptomic landscape throughout brain development. A total of 8766 genes were found to be differentially expressed (DEGs) along our time windows with a *p*-adjusted < 0.05. The largest transcriptomic changes occurred between E16 and P5 (7690 DEGs) and continued to P10 (8182 DEGs), while a smaller number of genes (5855 DEGs) changed between P5 and P10, reflecting a reduced scale of remodeling at later stages (Supplementary Figure S1B–D). A comprehensive examination of transcriptional profiles evidenced six distinct patterns throughout cortical development (Figure 1A). Genes in clusters 1, 2, and 3 are mostly low at E16 but increase at P5 and P10 (Figure 1A). These genes are involved in mitochondrial function, synapses, and neurotransmitter processing (Figure 1B). In contrast, genes in clusters 4, 5, and 6 are high at E16 but decrease by P5 or P10, and they are associated with nerve growth factor response, mRNA processing, and metabolism. Some pathways, including axonogenesis and neuron projection, resulted in enrichment across clusters 1, 3, and 5, indicating that they do not share a uniform expression profile throughout cortical development (Figure 1A,B). Interestingly, autophagy-related genes map primarily to clusters 2, 3, and 4 and show a marked increase at P10 (Figure 1B and Supplementary Figure S2). Positioned near terms such as locomotory behavior and forebrain development, these genes suggest a link between autophagy regulation, structural brain maturation, and emerging behavioral functions in the early postnatal period (Supplementary Figure S2).

**Figure 2:**
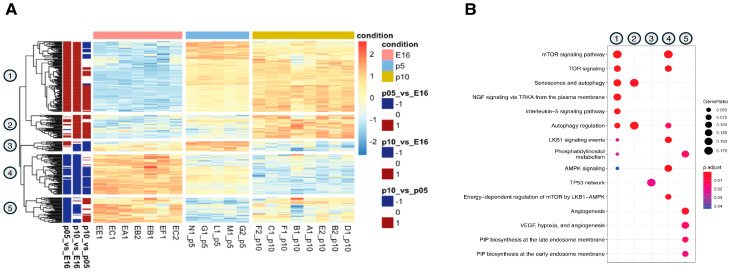
Autophagy is dynamically regulated during cortical development. (**A**) Heatmap showing the scaled expression of 274 autophagy genes divided into five clusters (numbered 1 to 5). It visually represents gene expression levels across different developmental stages: E16 (embryonic day 16.5), P5 (postnatal day 5), and P10 (postnatal day 10). (**B**) Dot plot of Gene Ontology (GO) enriched pathway analysis in developing cortices, indicating the top 30 most enriched pathways. The graph highlights that different biological processes drive the signature of each cluster.

Further investigation of the transcriptomic dataset identified 274 DEGs associated with autophagy (GO:0006914). Clustering of these genes revealed five distinct temporal profiles during cortical development, thus confirming that autophagy is regulated through subsets of genes active at specific developmental windows (Figure 2A). For instance, clusters 1 and 2 showed a trend of low expression at E16, which increased progressively at P5 and P10, indicating that these autophagy-related genes become more active during postnatal brain development, probably contributing to processes such as circuit maturation, neuronal connectivity, and synaptic plasticity.

In contrast, cluster 3 displayed a different pattern, with high expression at E16, a peak at P5, and a decline by P10. This trajectory implies a different functional role for these genes, likely becoming more critical during the transition from embryonic to early postnatal stages of brain maturation. Cluster 4 was characterized by genes that were highly expressed at E16, followed by a gradual decrease at P5 and P10, indicating a stronger involvement during early embryonic development. Finally, cluster 5 exhibited similar trends, with high levels at E16, a decrease at P5, and a modest increase at P10. This pattern suggests that these autophagy processes are highly active during embryonic development, temporarily downregulated during early postnatal stages, and re-engaged during later phases of neuronal maturation. Overall, these expression dynamics highlight the intricate regulation of autophagy throughout brain development. To gain insight into the biological processes of these clustered genes, we performed GO analysis of DEGs within each group. The results revealed that the mTOR signaling pathway is highly represented in clusters 1 and 4, while LKB1 and AMPK are more significantly enriched in cluster 4. NGF signaling via TRKA and interleukin-5 signaling were also enriched in cluster 1. Processes related to autophagy regulation and cellular senescence were prominently represented in clusters 1, 2, and 4. Cluster 3 was specifically enriched in DEGs involved in the TP53 network. Phosphatidylinositol metabolism and biosynthesis were mainly confined to cluster 5, along with pathways associated with angiogenesis.

**Figure 3:**
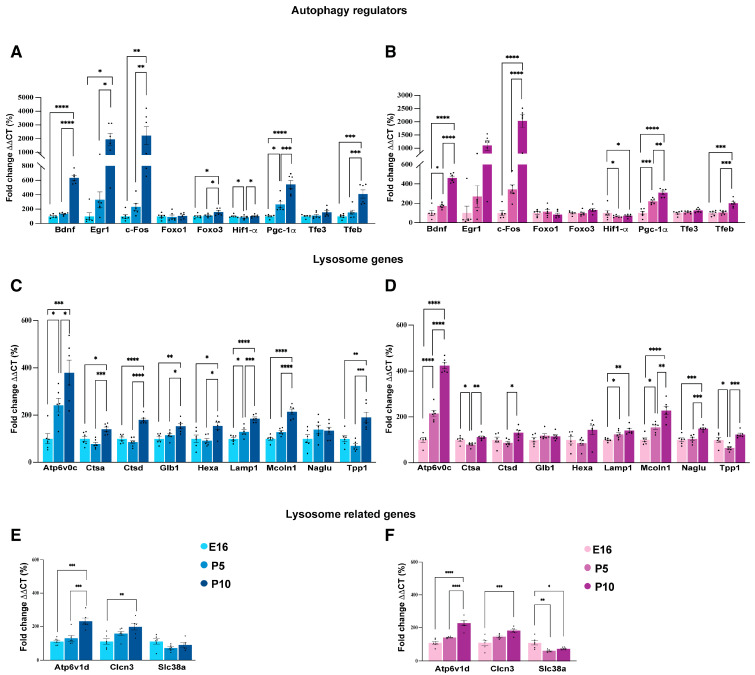
Lysosomal and autophagy gene expression during cortical development is sex independent. Transcriptomic profiles of the autophagy and lysosomal genes in developing male and female cortices. (**A**,**B**) Bar graph showing the mRNA levels of autophagy regulator genes in males at embryonic (E16, light blue), early postnatal (P5, medium blue), and later postnatal (P10, dark blue) stages (**A**) and females (E16, pink), early postnatal (P5, dark pink), and later postnatal (P10, violet) stages (**B**). (**C**,**D**) Developmental mRNA expression of lysosomal genes. Bar graphs illustrate the relative expression of representative lysosomal and lysosome-related genes in males (**C**,**E**) and females (**D**,**F**) across the same time points. Gene expression was normalized to the housekeeping gene cyclophilin and is presented as fold change relative to E16 levels (set as 100%). Data are shown as mean ± SEM from *n* = 6 biological replicates. Statistical significance was assessed using one-way ANOVA followed by Tukey’s post hoc test; * *p* < 0.05, ** *p* < 0.01, *** *p* < 0.001, **** *p* < 0.0001.

To better delineate the autophagy gene network dynamics during brain development, we performed a Gene Set Enrichment Analysis (GSEA) on a curated autophagy gene list as defined in the work of Bordi and colleagues [26]. In brief, they categorized 604 genes into seven main functional groups: (i) mTOR and upstream signaling pathways, (ii) core autophagy machinery, (iii) autophagy regulators, (iv) mitophagy-related genes, (v) docking and fusion components, (vi) lysosomal genes, and (vii) lysosomal-related genes. As depicted by the dot plot in Supplementary Figure S3A, the comparison P10 vs. E16 showed the most significant enrichment in gene sets related to lysosome and lysosome-related genes (indicated by large, dark red dots with asterisks), thus suggesting a substantial upregulation of these pathways between the embryonic stage (E16) and postnatal day 10 (P10). The comparison P10 vs. P5 displayed significant enrichment also for autophagy regulators, indicating that the autophagy machinery is more tightly controlled as the brain matures. In contrast, the P5 vs. E16 comparison manifested a much lower degree of enrichment. Overall, these data confirmed a temporal shift in the regulation of autophagy and lysosomal pathways during cortical development, becoming more active and well-regulated as the brain transitions from early embryonic stages to postnatal life, with the most significant changes occurring between P5 and P10. To confirm these findings, we measured the mRNA levels in cortices at E16, P5, and P10 collected from males and females separately (Figure 3). Real-time qPCR of autophagy regulator genes showed that the expression of *c-Fos*, *Bdnf*, and *Egr1* increases from E16 to P10. Similarly, *Pgc1α* and Tfeb displayed a notable upregulation at P10 compared to the earlier stages. In contrast, *Foxo1*, *Foxo3*, *Hif1α*, and *Tfe3* exhibited little to no change across all time points (Figure 3A,B). Lysosomal and related genes manifested an overall higher expression at P10 compared to E16 and P5, as illustrated in Figure 3C–F. Although the group of “core autophagic genes” was not statistically significant in the GSEA (Supplementary Figure S3A), we decided to test the mRNA levels of some key components. Indeed, the mRNA levels of *Atg9a* and *Sqstm1* increased at P10 compared to P5 and E16 (Supplementary Figure S3B,C). The expression of *Atg16l*, *Gabarapl1*, and *Lc3* decreased between E16 and P5, with either no change or a slight increase observed at P10 (Supplementary Figure S3B,C). Conversely, Ulk1 showed the opposite pattern, significantly decreasing from the embryonic to the postnatal stage (Supplementary Figure S3B,C). Collectively, these results indicate a developmental shift in gene expression, where the increase in autophagy and lysosomal function genes observed in both male and female mice from E16 to P10 likely reflects the heightened metabolic and cellular housekeeping demands of the maturing brain that coincide with the synaptogenesis window.

### 3.2. Mapping the Correlation Between Autophagy and Synaptic Architecture in the Postnatal Cortex

To investigate the functional relationship between autophagy and synaptic function, we conducted a gene co-expression analysis. Specifically, we selected biological processes significantly associated with either autophagy or synaptic activity and calculated the mean pairwise correlation among genes annotated to these processes.

As indicated by the heatmap, a strong correlation emerged between these two pathways (Pearson’s correlation coefficients between 0.90 and 0.99, correlation test *p*-values between 4.4 × 10^−9^ and 5.6 × 10^−25^) (Supplementary Figure S4A,B), thus reinforcing the concept that autophagy is not merely a cellular cleanup mechanism but may also play a crucial role in shaping the complex architecture of synapses. Guided by transcriptomic data, we set out to characterize the dynamics of autophagic proteins associated with the synaptic compartment. To this end, we isolated crude synaptosomes, which are artificial organelles retaining essential synaptic components, from the cortices of WT mice at three postnatal developmental stages (P5, P10, and P20), which span the onset, progression, and maturation of synaptogenesis, a process that occurs predominantly after birth. We first validated the enrichment of synaptosomal fractions isolated from brain homogenates by analyzing the expression of markers associated with different subcellular compartments (Supplementary Figure S5). Immunoblot analysis revealed a predominant expression of Synapsin 1, as expected, in the P2′ fraction. In contrast, the endoplasmic reticulum marker calnexin and the cytosolic protein GAPDH were minimally detected in the same fraction. Notably, the mitochondrial marker TIM23 showed a relatively higher abundance (Supplementary Figure S5), consistent with previous reports [19,20]. This likely reflects the retention of mitochondria in crude synaptosomal (P2′) fractions, a known technical feature due to their tight association with presynaptic terminals. To unravel the protein contents that shape these structures during early stages of development, we subjected the cortical purified synaptosomes to advanced mass spectrometry-based proteomics analysis. Three to four independent biological samples per time point were analyzed using a label-free quantitative approach. PCA indicated a clear separation among the three different time points (Supplementary Figure S6A), suggesting that the proteome is extremely different during brain development. A total of 1035 proteins were identified by filtering for those with at least a 50% quantification rate in one condition; however, the statistically significantly differentially expressed proteins (DEPs) were 514.

Compared with P5, P10 and P20 showed progressively more deregulated proteins, with the largest changes occurring between P10 and P20, reflecting extensive proteomic remodeling and a major shift in synaptic protein composition (Supplementary Figure S6B–E; Table 2).

**Figure 4:**
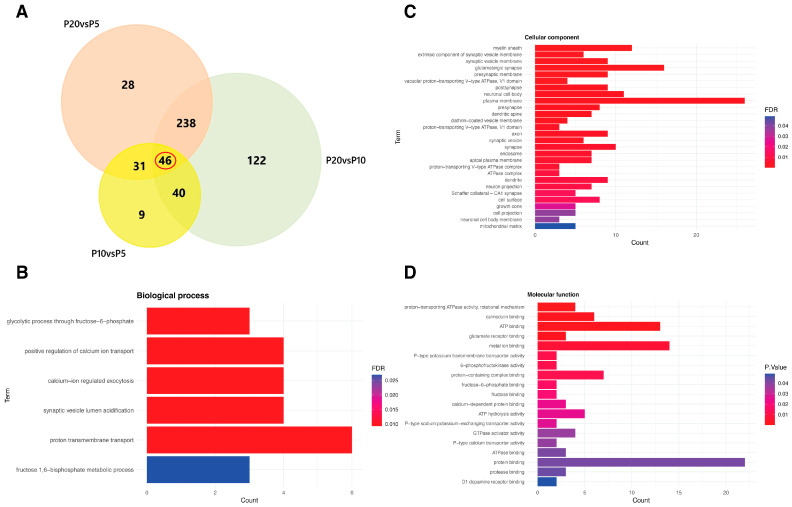
Comparative analysis of deregulated synaptic proteins across development. (**A**) Venn diagram indicating the overlap of proteins found deregulated among the three comparisons: P20 vs. P10, P20 vs. P5 and P10 vs. P5. The red circle highlights the core set of genes that are significantly changed across all three experimental conditions. (**B**–**D**) Enriched categories of deregulated proteins are represented by the bar graph for each comparison. The vertical axis shows the top GO terms, while the horizontal axis displays the protein counts belonging to each category with FDR < 0.05 in (**B**,**C**) and *p* adj < 0.05 in (**D**).

When comparing samples to the early P5 stage, P10 exhibited 126 deregulated proteins (FDR-adjusted *p*-value < 0.05), with 59 showing increased expression (upregulated) and 67 showing decreased expression (downregulated) (Supplementary Figure S6B,C). As development progressed to P20, we observed a substantially greater number of changes relative to P5, with a total of 343 DEPs (293 upregulated and 50 downregulated proteins) (Supplementary Figure S6B,D). Remarkably, a direct comparison between P20 and P10 revealed extensive proteomic remodeling, with 446 proteins undergoing deregulation (398 upregulated and 48 downregulated proteins), suggesting a major shift in the proteomic composition of synaptic structures occurring between P10 and P20 (Supplementary Figure S6B,E; Table 2). Gene ontology analysis consistently identified synaptic compartments as the most prominent across all three comparisons (Supplementary Figure S6F–H). Among these, 46 deregulated proteins were shared (Figure 4A) and were enriched in synaptic-related cellular compartments (Figure 4C) as well as in processes involving glycolysis, calcium transport, and vesicle acidification (Figure 4B–D).

A deeper investigation of changes in synaptic proteins revealed a total of 181 deregulated proteins in developing cortical synaptosomes (Table 2).

**Figure 5:**
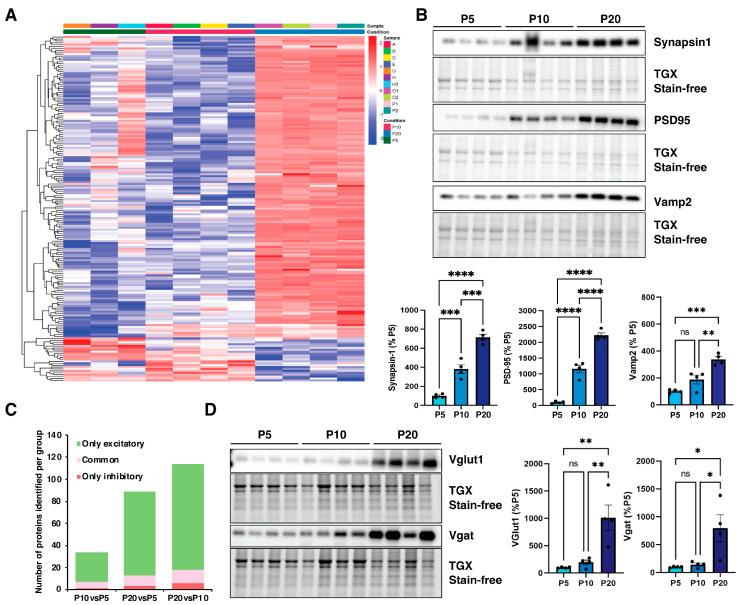
Synaptic proteins are mostly upregulated in developing wild-type mice cortices. (**A**) Heatmap of 181 differentially expressed synaptic proteins. Red color indicates high expression; blue indicates low expression. (**B**) Protein expression levels of Synapsin1, VAMP2 (pre-synaptic markers), and PSD95 (post-synaptic compartment marker) were measured by Western blot at three stages of cortical development: P5, P10, and P20. (**C**) Bar graph showing the number of excitatory, inhibitory, and shared synaptic proteins in each comparison. (**D**) Immunoblotting showing the expression levels of V-GLUT1 (marker of glutamatergic synapses) and V-GAT (marker for inhibitory synapses) during cortical development (P5, P10, and P20). The protein levels were normalized to the total protein lysates. Data are shown as mean ± SEM (*n* = 4 per time point). One-way ANOVA followed by Tukey’s test. * *p* < 0.05, ** *p* < 0.01, *** *p* < 0.001 and **** *p* < 0.0001; ns = not significant.

The heatmap of synaptic proteins showed that the majority of these are upregulated at P20 compared to earlier developmental stages (Figure 5A), in line with previous reports [27,28,29].

Moreover, hierarchical clustering identified two distinct groups: cluster 1 comprises proteins selectively upregulated at P20, whereas cluster 2 includes proteins that are predominantly downregulated at later stages of development. (Figure 5A). To confirm these dynamics, we measured the expression levels of key synaptic proteins across the selected time periods. As shown by the Western blot, pre-synaptic synapsin 1 exhibited a progressive and statistically significant increase in expression levels. Specifically, an upregulation was observed from P5 to P10 (*** *p* < 0.001) followed by a further elevation from P10 to P20 (** *p* < 0.01) (Figure 5B). This pattern is consistent with the expansion in synaptic density and the increasing complexity of presynaptic terminals during cortical maturation. Similarly, the vesicle-associated membrane protein 2 (VAMP2) increased its expression during the developmental stages (Figure 5B). Finally, the post-synaptic marker PSD95 (post-synaptic density protein 95) displayed a significant upregulation between P5 and P10 (**** *p* < 0.0001) and between P10 and P20 (**** *p* < 0.0001) (Figure 5B), reflecting the maturation and organization of the post-synaptic density in conjunction with the formation and strengthening of synapses. Notably, a subset (*n* = 159) of the deregulated proteins was annotated in SynGO and subdivided into six categories (Supplementary Figure S7A). The majority of these proteins are presynaptic or involved in synaptic organization (Supplementary Figure S7B). We further characterized the nature of synaptosomes along cortical development by determining the changes in excitatory and inhibitory synaptic markers. Among the significantly altered proteins, a total of 106 were ascribed to glutamatergic synapses exclusively, while 7 were uniquely defined as GABA-ergic; 14 proteins could function at both excitatory and inhibitory synapses (Figure 5C). As evidenced in Figure 5D, the expression of V-GLUT1 (Vesicular Glutamate Transporter), a key indicator of glutamatergic synapses, was augmented from P10 to P20 (** *p* < 0.01). Similarly, V-GAT (anti-vesicular GABA transporter), a marker for inhibitory synapses, showed a significant upregulation from P10 to P20 (Figure 5D). This trend suggests a proliferation and maturation of inhibitory and excitatory synapses during the later stage of brain development. Overall, these data are consistent with the well-known phase of synaptogenesis and synaptic maturation that characterizes the mammalian brain [30,31,32].

Finally, we examined whether autophagic proteins were enriched in developing cortical synaptosomes. With this aim, we interrogated the dataset using the list reported by Bordi and colleagues [26], as previously done for the transcriptomic analyses. Interestingly, we identified 55 autophagic–lysosomal proteins, 37 of which were significantly deregulated (Table 3).

**Figure 6:**
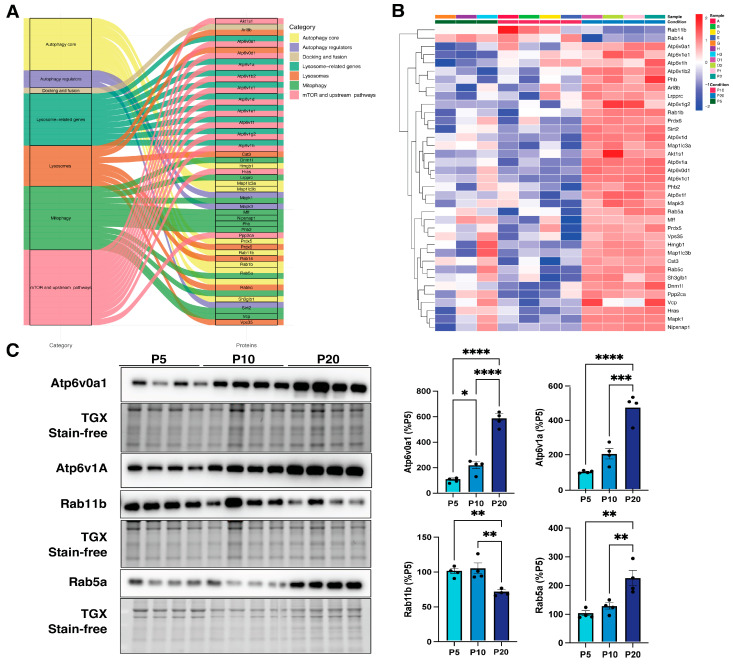
Autophagy and lysosomal proteins at the synaptic site are predominantly upregulated in maturing wild-type cortices. (**A**) Alluvial plot showing the different categories of the deregulated autophagy-lysosomal proteins across the developmental maturation, as found in synaptosomes. (**B**) Heatmap illustrating the expression profile of deregulated autophagy-lysosomal proteins purified from synaptosomes across cortical developmental stages. Red color indicates high expression; blue indicates low expression. (**C**) Immunoblot from synaptosomal lysates of WT cortices at different stages of postnatal development (P5, P10, and P20). Atp6v0a1, Atp6v1a, Rab5a, and Rab11 signal intensities were quantified by densitometric analysis. Protein levels were normalized to total protein using stain-free technology. Data are shown as mean ± SEM (*n* = 4 per time point), expressed as a percentage of the P5 mean. Statistical significance was assessed using one-way ANOVA followed by Tukey’s post hoc test; * *p* < 0.05, ** *p* < 0.01, *** *p* < 0.001 and **** *p* < 0.0001.

This targeted approach allowed us to specifically focus on autophagy–lysosomal components, minimizing potential confounding signals from other synaptic proteins or residual mitochondrial proteins. Interestingly, of the 55 autophagy–lysosomal proteins identified, 37 were significantly deregulated across development and belong to different functional categories (Table 3, Figure 6A). Similar to synaptic proteins, their expression increased during brain development (Figure 6B). Notably, several ATP6 synthase subunits showed marked upregulation, suggesting enhanced synaptic acidification, which is consistent with the primary role of synaptic vesicles in neurotransmitter loading and release during neuronal transmission [33]. Western blot analysis confirmed this expression trend for ATP6V1A and ATP6V0A1 in purified synaptosomes across developmental stages (Figure 6C).

In addition, diverse Rab proteins were found to be markedly deregulated. Notably, Rab1b, Rab5a, and Rab5c showed increased expression as the cortex matured, whereas Rab11b and Rab14 exhibited the opposite trend (Figure 6B). Changes in protein levels were further validated for Rab5a and Rab11b by immunoblot measurements (Figure 6C). Together, these findings demonstrate a dynamic and developmentally regulated remodeling of the autophagy–lysosomal machinery at cortical synapses. The coordinated upregulation of lysosomal ATP6 synthase subunits and specific Rab GTPases suggests a progressive enhancement of autophagic and endo-lysosomal trafficking capacity during postnatal brain development. This maturation of autophagy-related pathways at synaptic compartments is likely to support synaptic growth, remodeling, and circuit refinement, underscoring a previously underappreciated contribution of autophagy–lysosomal processes to synaptic maturation.

## 4. Discussion

Over the past decades, the importance of regulatory gene networks in controlling cell metabolism has become evident in every aspect of neuronal functions [34,35]. Despite significant advances in our understanding of the transcriptional regulation of autophagy, the broader gene network orchestrating this pathway in developing neurons remains largely unexplored. Identifying which specific autophagy genes are activated or silenced during distinct stages of brain development could provide valuable insight for designing therapeutic strategies, particularly for neurodevelopmental disorders.

Overall, our transcriptomic analysis indicates that cortical development is driven by an integrated network of pathways, with autophagy emerging as a dynamically regulated component. Accordingly, temporal clustering revealed distinct developmental regulation of autophagy-related genes (GO:0006914) across cortical maturation. Genes in clusters 1 and 2 were progressively upregulated from E16 to P5 and P10, indicating increased postnatal activation of autophagy-related processes associated with synaptic maturation, neuronal connectivity, and plasticity. These clusters were enriched for mTOR signaling, NGF–TRKA and interleukin-5 signaling, as well as pathways regulating autophagy and cellular senescence, suggesting their predominant deregulation during postnatal development. In contrast, cluster 3 genes peaked during the embryonic-to-early postnatal transition (E16–P5) and were enriched for TP53-associated pathways, indicating a stage-specific role during early brain maturation. Clusters 4 and 5 were characterized by high embryonic expression followed by postnatal downregulation, highlighting pathways predominantly active at early developmental stages. Cluster 4 was enriched for mTOR, LKB1, and AMPK signaling, implicating energy sensing and autophagy regulation during embryogenesis, whereas cluster 5 was associated with phosphatidylinositol metabolism and angiogenesis, suggesting transient engagement during embryonic development with partial reactivation at later stages.

To narrow our analysis, we followed the expression of a subset of autophagy and lysosomal genes during cortical development in both males and females. Notably, we observed an overall increase in these genes that was consistent across sexes, suggesting minimal sex-specific effects during this developmental window. While sex-specific differences in autophagy were not systematically assessed in the RNA-seq and proteomics datasets due to pooling of samples, qPCR validation in males and females separately confirmed comparable expression trends, in line with previous reports [36]. Moreover, we observed the decreasing expression of *Ulk1* from the embryonic stage (E16) to the postnatal stage (P10) in the cortex, thus suggesting a functional shift from processes that heavily rely on high ULK1 activity to those that require its downregulation, particularly during a period of intense neuronal differentiation, synaptogenesis, and circuit maturation.

Growing evidence indicates that autophagy plays an active role in synaptic development and function, extending beyond its canonical role in cellular homeostasis to directly regulate synaptic remodeling and plasticity. Consistent with this notion, our transcriptomic profiling revealed a strong association between autophagy-related genes and synaptic pathways during cortical development. Notably, a targeted interrogation of cortical synaptosomal proteomes using a curated autophagy gene list [26] uncovered a substantial enrichment of autophagy–lysosomal proteins that increased in abundance over developmental time. In particular, the upregulation of multiple ATP6 synthase subunits suggests a progressive enhancement of synaptic acidification, a feature previously linked to synaptic maturation and circuit refinement. Moreover, the differential regulation of Rab GTPases involved in endo-lysosomal trafficking further supports the idea that precise spatial and temporal control of autophagy-related pathways is required for synaptic development. While our data provide a comprehensive framework that highlights autophagy as a potentially important regulator of synaptic maturation in the mammalian cortex, we acknowledge that functional perturbation experiments will be necessary to directly test causality. Nevertheless, these observations are consistent with recent studies demonstrating that synaptic autophagy contributes to the maturation and remodeling of neuronal circuits and further support the view that autophagy represents a dynamic and integral component of synaptic development in the mammalian cortex. In conclusion, our findings reveal that autophagy during brain development is tightly integrated with metabolic signaling, synaptic function, and cell fate decisions, underscoring its multifaceted role beyond cellular clearance. The coordinated regulation of autophagy–lysosomal components at synaptic sites points out their contribution to synaptic maturation, remodeling, and circuit refinement. Importantly, synaptic dysfunction is a hallmark of several neurodevelopmental disorders (NDDs), including autism spectrum disorders, epilepsy, and intellectual disabilities, which are frequently associated with impaired synaptic plasticity, altered neurotransmitter receptor composition, and dysregulation of genes governing synapse formation and maintenance. Our findings suggest that precise temporal regulation of autophagy–lysosomal pathways is critical for proper synaptic maturation and circuit refinement and that disruptions in these pathways during key developmental windows could contribute to the synaptic deficits observed in NDDs, such as autism spectrum disorders, fragile X syndrome, and Rett syndrome, in which alterations in autophagy–lysosomal signaling and synaptic function have been reported [10,13,37,38]. By systematically mapping the transcriptional and proteomic landscape of autophagy in developing cortical neurons, we provide a framework for identifying candidate pathways and proteins that may be affected in pathological contexts. Ultimately, this knowledge could guide future studies in experimental disease models and inform the design of therapeutic interventions aimed at restoring normal synaptic function through modulation of autophagy–lysosomal pathways.

## Figures and Tables

**Table 1:** Primers used for real-time qPCR validation of 28 genes representing the connected network of autophagy–lysosomal pathways during cortical development.

Gene Name	Primer Forward 5′-3′	Primer Reverse 5′-3′
*Atg9a*	GCGTTGCTGGTCACTCTATG	CAGGATAGAGCCAGCGAAGA
*Atg16l1*	TCGCCTCAATGCAGAGAATG	GAGGTTCCTTTGCTGCTTCT
*Atp6v0c*	CAGAGCTGATCATGAAGTCCATC	GAAAACTCCTGTAGATGATG
*Atp6v1d*	GGATTGAACGCACCCTTG	GCTGCTCTCCGCCGCTC
*Bdnf*	AAGTCTGCATTACATTCCTCGA	GTTTTCTGAAAGAGCGACAGTT
*C-fos*	AGAGCGCCCCATCCTTAC	GCAGCCATCTTATTCCGTTC
*Clcn3*	CCTGGCTGCTGATGTTATGA	TGAGGGCAAATCCCACTAAT
*Ctsa*	TTCTGATCCAGCCAGATGGTG	TACAGCACGTTGGCAATCAGG
*Ctsd*	CGTCCTTTGACATCCACTACGG	TGGAACCGATACAGTGTCCTGG
*Cyclophilin*	GGCAAATGCTGGACCAAACACAA	GTAAAATGCCCGCAAGTCAAAAG
*Egr1*	CCTTCAATCCTCAAGGGAGC	AACCGAGTCGTTTGGCTGGGA
*Foxo1*	GTGGATGGTGAAGAGCGTGCCC	GCTGTGAAGGGACAGATTGTGGCG
*Foxo3*	AAGGGCGACAGCAACAGCTCTG	CATGAAGCGGCTGTGCAGGGAC
*Gabarapl1*	CAAAGAGGAGCATCCGTTCGAG	TTGTCCAGGTCTCCTATCCGAG
*Glb1*	AAATGGCTGGCAGTCCTTCTG	ACCTGCACGGTTATGATCGGT
*Hexa*	CTACATCCAGACGCTGCTGGAC	TACTGGCATTTCTTCCCGCCAC
*Hif1-α*	GCTGAAGACACAGAGGCAAA	TACTTGGAGGGCTTGGAGAA
*Lamp1*	CCTACGAGACTGCGAATGGT	CCACAAGAACTGCCATTTTTC
*Map1lc3b*	GCTTGCAGCTCAATGCTAAC	CCTGCGAGGCATAAACCATGTA
*Mcol1*	GCGCCTATGACACCATCAA	TATCCTGGCACTGCTCGAT
*Naglu*	TCCAACAGCACGAGTTTGAG	CTGCGATGGCTAATCTGTCA
*Pgc1-α*	GAATCAAGCCACTACAGACACGC	CATCCCTCTTGAGCCTTTCGTG
*Slc38a9*	TTGAAAGCGAGGGAAATGATGGTC	ATGGGAATGAGGGTCACTGAGAAG
*Sqstm1*	GAAGCTGCCCTATACCCACA	TGGGAGAGGGACTCAATCAG
*Tfe3*	AGGATCAAAGAGCTGGGCAC	CCGGCTCTCCAGGTCTTTG
*Tfeb*	GTCATTGACAACATTATGCGCC	GCGTGTTAGGCATCTTGCATCT
*Tpp1*	CCCCTCATGTGGATTTTGTGG	TGGTTCTGGACGTTGTCTTGG
*Ulk1*	TGGAGACCTGGCTGACTACCTGC	TGGATGATGCCCTTGCTGTGCA

**Table 2:** Summary of significantly deregulated total and synaptic proteins in the developing mouse cortex.

	Significant (FDR < 0.05)			
	**N° of proteins**	**P10 vs. P5**	**P20 vs. P10**	**P20 vs. P5**
Total	514	126	446	343
Synaptic	181	31	75	51
%	35	25	17	15

**Table 3:** Summary of significantly deregulated total and autophagic proteins in the developing mouse cortex.

	Significant (FDR < 0.05)			
	**N° of proteins**	**P10 vs. P5**	**P20 vs. P10**	**P20 vs. P5**
Total	514	126	446	343
Autophagy-Lysosomal	37	6	24	15
%	7	5	5	4

## Data Availability

The datasets produced in this study are available in the following databases: RNA-Seq data: Gene Expression Omnibus # GSE319437. Proteomic data: Data are available via ProteomeXchange with identifier PXD074614.

## Supplementary Materials

The following supporting information can be downloaded at https://www.mdpi.com/article/10.3390/biomedicines14040812/s1, Figure S1: Transcriptomic profiling of cortical development; Figure S2: Gene Ontology (GO) network showing autophagy and linked pathways. Figure S3: Enrichment analysis of autophagy and lysosomal pathways. Figure S4: Autophagy and synaptic-related processes are co-regulated during cortical maturation. Figure S5: Synaptosomal enrichment analysis. Figure S6: Proteomic profiling of developing cortical synaptosomes. Figure S7: Functional and spatial distribution of synaptic proteins.

## Abbreviations

The following abbreviations are used in this manuscript:

mTOR	Mechanistic Target of Rapamycin
AMPK	AMP-Activated Protein Kinase
ULK1	Unc-51-Like Autophagy Activating Kinase 1
LKB1	Liver Kinase B1
SEM	Standard Error of the Mean
NGF	Nerve Growth Factor
TRKA	Tyrosine Kinase A
PICK1	Protein Interacting with PRKCA 1
SHANK3	SH3 And Multiple Ankyrin Repeat Domains 3
TIM23 or TIMM23	Translocase of Inner Mitochondrial Membrane 23
