# Supplementary material for: Combined Transcriptomic and Proteomic Profiling Uncovers Developmental Dynamics of Autophagy in the Cortex
Source: Biomedicines. 2026 Apr 2;14(4):812. doi: 10.3390/biomedicines14040812 (PMC13113489; doi:10.3390/biomedicines14040812)
Supplement: Supplementary file 1 [file biomedicines-14-00812-s001.zip › biomedicines-4184348-supplementary.pdf]

Supplementary Figure S1

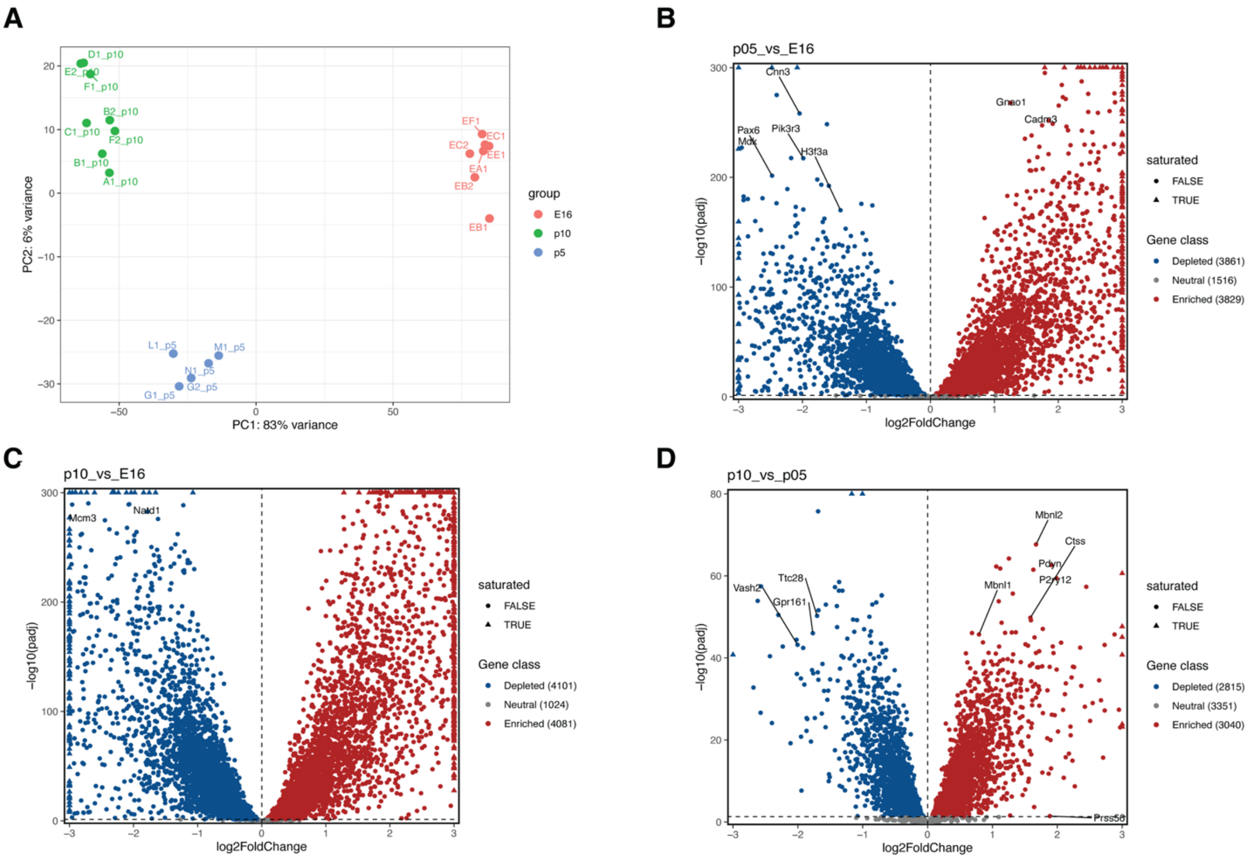

Supplementary Figure 1. Transcriptomic profiling of cortical development. **(A)** Principal component analysis (PCA) plot of sequenced samples at E16 (*n* = 7), P5 (*n* = 5) and P10 (*n* = 8) cortices. The percentage of variance explained by the first (PC1) and second (PC2) components is indicated. Samples from different time points are clearly separated, highlighting stage-specific transcriptomic signatures during cortical development. **(B-D)** Volcano plot showing differentially expressed genes (DEGs) for the indicated comparison: **(B)** P05 vs E16, **(C)** P10 vs E16 and **(D)** P10 vs P5. The number of DEGs with a Log2FoldChange < 0 (down-regulated genes) or > 0 (upregulated genes) is also indicated, considering an adjusted p-value (*p.adj*) < 0.05. In detail, when comparing the transcriptomic profiles to the early E16 stage, P5 exhibited 7690 deregulated genes, with 3829 showing increased expression (up-regulated) and 3861 showing decreased expression

(down-regulated) (Supplementary Figure 1B). As development progressed to P10, we observed a total of 8182 DEGs (4081 up-regulated and 4101 down-regulated genes) (Supplementary Figure 1C). Remarkably, a direct comparison between P10 and P5 revealed a less extensive genetic remodeling, with 5855 genes undergoing deregulation (3040 up-regulated and 2815 down-regulated genes at P10) (Supplementary Figure 1D).

Supplementary Figure S2

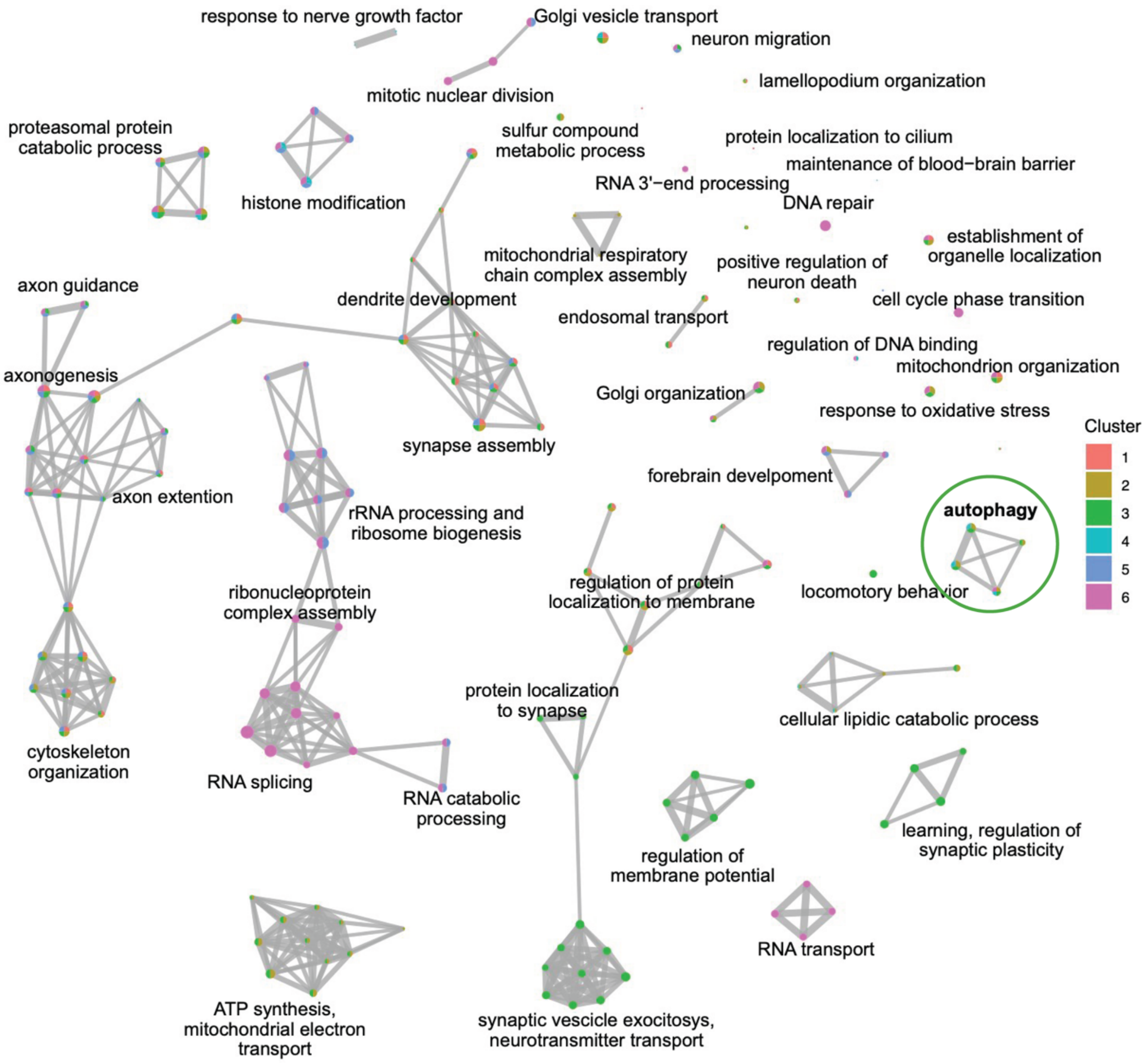

Supplementary Figure 2. Gene Ontology (GO) network showing autophagy and linked pathways. Functional network analysis displaying the relationships between various biological processes and autophagy. Each node (circle) represents a GO term, and the lines connecting them indicate a significant functional relationship or shared genes. The nodes are color-coded based on which cluster they belong to, as indicated by the legend. The cluster highlighted by a green oval contains terms related to autophagy.

Supplementary Figure S3

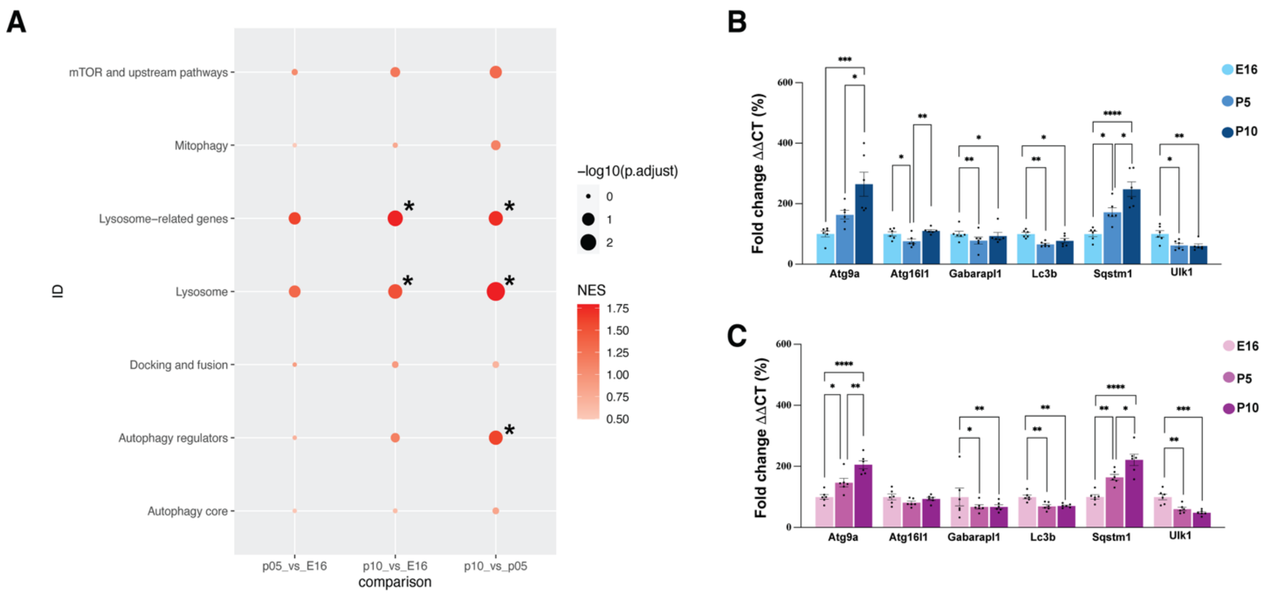

Supplementary Figure 3. Enrichment analysis of autophagy and lysosomal pathways. **(A)** The dot plot displays the results of a Gene Set Enrichment Analysis (GSEA) for specific autophagy, lysosomal, and associated regulatory pathways (y-axis) across three developmental comparisons (x-axis). **(B and C)** Relative mRNA levels of core autophagic genes are shown in males **(B)** and females **(C)** WT mice cortices at E16, P5 and P10. Gene expression was normalized to the

housekeeping gene cyclophilin and is presented as fold change relative to E16 levels (set as 100%). Data are shown as mean ± SEM from n = 6 biological replicates. Statistical significance was assessed using one-way ANOVA followed by Tukey's post hoc test, with $p < 0.05$ considered significant.

Supplementary Figure S4

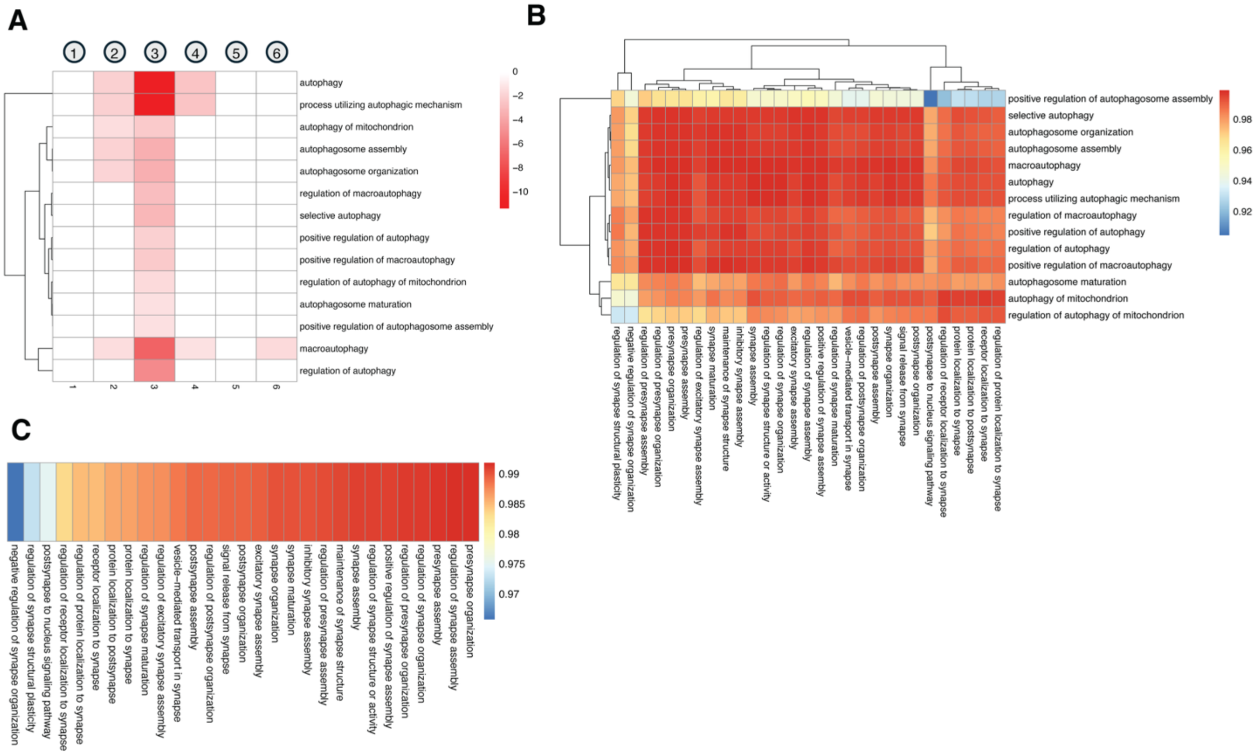

Supplementary Figure 4. Autophagy and synaptic related processes are co-regulated during cortical maturation. **(A)** Co-expression analysis of genes associated with enriched autophagy and synaptic processes in the six clusters (numbered 1 to 6) highlighted in Figure 1. The color intensity represents the degree of overlap or correlation between autophagy and synaptic pathways during cortical development. **(B-C)** Heatmap illustrating the similarity in expression patterns among different autophagy and synapse-related gene ontology (GO) categories. Higher color intensity indicates strong co-expression.

Supplementary Figure S5

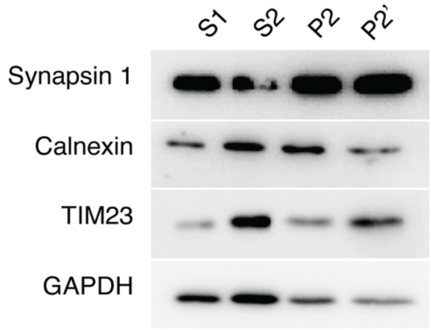

Supplementary Figure 5. Synaptosomal enrichment analysis. The enrichment of the synaptosomal fraction was assessed by measuring the expression levels of Synapsin 1 in the P2' fraction. The purity of these fractions was further evaluated by quantifying the abundance of two cellular markers: Calnexin, representing endoplasmic reticulum (ER) and TIM23, representing mitochondria. As shown, the P2' fraction is highly enriched in Synapsin1, whereas Calnexin and TIM23 are present at low levels, confirming the specificity of the synaptosomal preparation.

Supplementary Figure S6

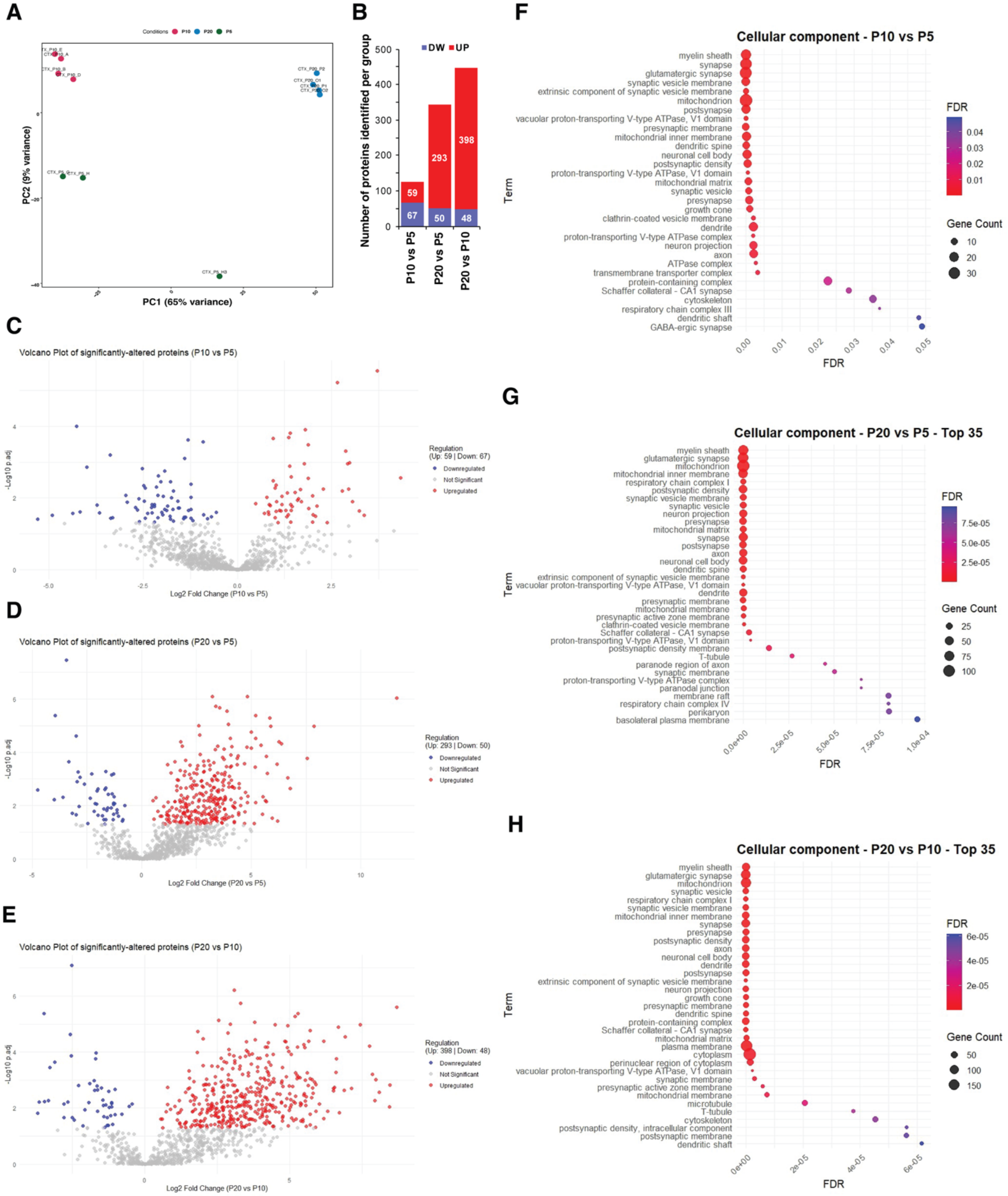

Supplementary Figure 6. Proteomic profiling of developing cortical synaptosomes. **(A)** Principal component analysis (PCA) plot of sequenced samples at P5 ($n = 3$), P10 ($n = 4$) and P20 ($n = 4$) cortices. The percentage of variance explained by the first (PC1) and second (PC2) components is indicated. Samples from different time points are clearly separated, highlighting stage-specific proteomic signatures during cortical development. **(B)** Bar diagram reporting the number of differentially expressed proteins (DEPs) among the three comparisons. **(C-E)** Volcano plot representing the number of deregulated proteins in each comparison (**C**, P10 vs P5; **D**, P20 vs P5 and **E**, P20 vs P10), with an adjusted p-value (*p.adj*) < 0.05. **(F-H)** Gene Ontology (GO) enrichment analysis showing the most significantly enriched cellular component categories in each comparison. Categories are ranked based on statistical relevance, while the dot size corresponds to the number of differentially expressed proteins associated with that specific cellular component term. On the x axis is indicated the statistical significance of the enrichment for each term.

Supplemenary Figure S7

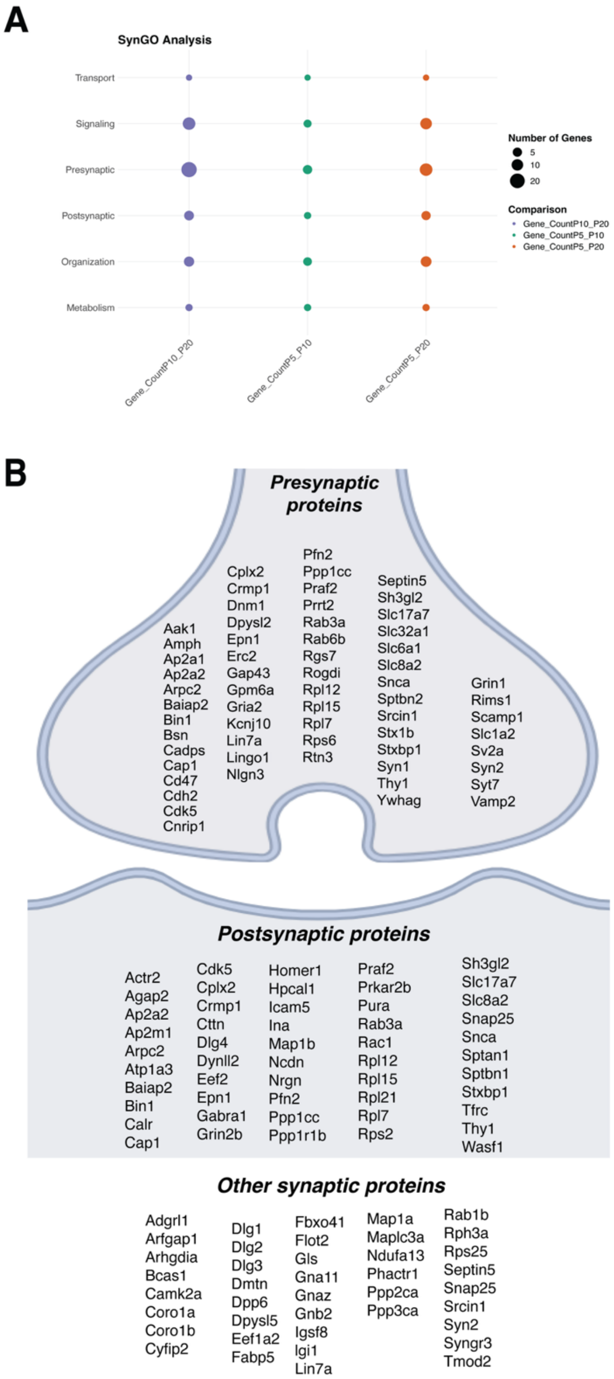

Supplementary Figure 7. Functional and spatial distribution of synaptic proteins. **(A)** SynGO analysis indicating the six categories and number of gene enriched in each term and comparison. **(B)** Topology of synaptic protein identified in purified cortical synaptosomes.
